# The importance of interdisciplinary collaboration in advanced therapy of odontogenic cysts: A 31 Month follow-up case report

**DOI:** 10.1016/j.heliyon.2024.e37587

**Published:** 2024-09-12

**Authors:** Kinga Bérczy, Csilla Erdei, Hajnalka Rajnai, Gergely Hriczó-Koperdák, Árpád Fancsaly-Joób, Noémi Kovács

**Affiliations:** a Department of Oro-Maxillofacial Surgery and Stomatology, Semmelweis University, Faculty of Dentistry, Hungary; b Department of General Dental Preclinical Practice, Semmelweis University, Faculty of Dentistry, Hungary; c Department of Pathology and Experimental Cancer Research, Semmelweis University, Faculty of Medicine, Hungary; d Dentop Dentistry and Oral Surgery, Hungary

## Abstract

In this case report, we present the treatment of a 39-year-old male patient with a bilateral maxilla cyst diagnosed as an additional finding ont he X-ray. Both conservative dentistry treatments and oral surgical procedures were carried out using state-of-the-art materials and equipment, and in close collaboration with the other dental specialists. Endodontic treatment of the remaining teeth was performed before the oral surgery treatment. The root canal fillings were made using bioceramic-based root canal sealer. Cystectomy was then performed on both sides and the bone cavities were filled with platelet-rich fibrin (PRF). In the 4th month X-ray after the operation, radiological images showed bone regeneration. After 31 months, the periapical region is intact on the X-ray, the function of the root canal treated teeth has been preserved and the patient is free of complaints. With the chosen therapy we achieved a complication-free, long-term successful result in a time-efficient manner.

## 1 Introduction

The therapeutic options for odontogenic cysts have a long history. The two most important surgical therapeutic options, cystostomy and cystectomy, were described by the Polish professor *Carl Franz Maria Partsch* in 1892 and 1910 respectively [1]. Both treatment methods are still the basis of surgical procedures today [2]. The prevalence of odontogenic cysts is between 14 and 16 % based on literature data and about 50–60 % of these are radicular cysts [3]. Radicular cysts have previously been classified as inflammatory cysts [4], but according to the latest WHO classification of odontogenic cysts, no separate subgroups are distinguished [5]. In many cases, in addition to surgical therapy, the treatment of odontogenic cysts also includes the preservation of the non-vital tooth that caused the lesion or was covered by the cyst. In this way, we can reduce the extent of the trauma for the patient and the need for subsequent prosthetic rehabilitation. In such cases, close cooperation between specialists in endodontics and oral surgery is warranted when planning and carrying out treatment. In both fields, there has been a steady and rapid evolution of the materials used, with the treatment of odontogenic cysts being no exception. In today's modern dentistry, interdisciplinary collaboration is essential to provide patients with state-of-the-art care that can reduce recovery time and improve long-term prognosis. In our work, we present the possibility of treating radicular cysts involving both sides of the maxilla using state-of-the-art materials, emphasizing the importance of close cooperation between different specializations.

## 2 Case report

**Fig. 1:**
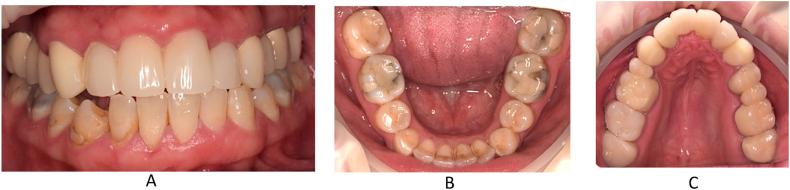
Preoperativ imgages: A. intercuspal position (ICP), B. lower jaw, C. upper jaw.

**Fig. 2:**
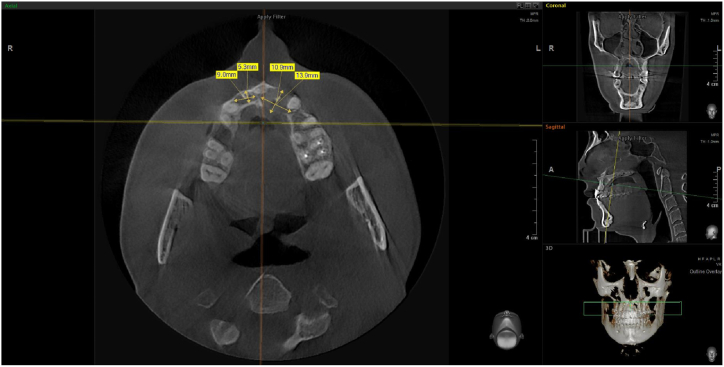
Horizontal cross section of the maxilla and the sizes of the cysts.

**Fig. 3:**
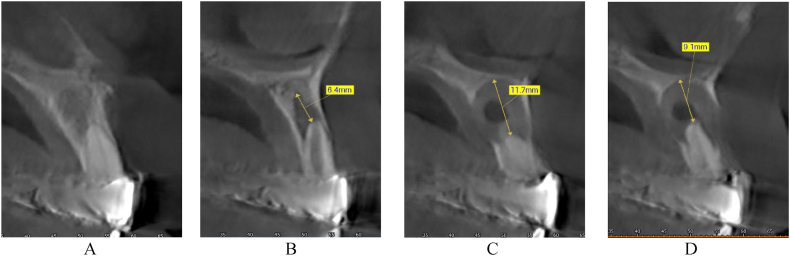
Axial cross section of the 11 tooth (A), the 12 tooth (B), the 21 tooth (c) and the 22 tooth (D).

In this case, we present the treatment of a 39-year-old male patient. His general medical history includes two catheter cardiac ablations (2008, 2013) and a cholecystectomy. (2015). The patient has no known social habits, such as smoking or regular alcohol consumption. The patient visited the Department of Oro-Maxillofacial Surgery and Stomatology at Semmelweis University in February 2021. According to him, a few weeks earlier, his dentist had noticed a lesion in the front region of the maxilla during his annual dental check-up (on panoramic x-ray), and had also performed a cone beam computed tomography (CBCT) scan. The patient was free of complaints at the time of the examination and reported no previous problems in the region. While taking his dental history, he mentioned that he had fallen down the stairs when he was 7–8 years old and had hit his face in this area. The soft tissue injuries were treated with a suture, no dental intervention was performed (no documentation of the case is available). This traumatic injury is thought to be the cause of the current maxillary cyst. His referring dentist removed the previous metal-ceramic splint from teeth 12, 11, 21 and 22 and placed a temporary polymethyl methacrylate (PMMA) splint. For the purpose of this case study, this condition is considered the baseline condition (Fig. 1). In the analysis of the CBCT image taken, we saw separate maxillary cysts in the periapical region of tooth 12 measuring approximately 5 × 9 mm (Fig. 2, Fig. 3A) and in the region of teeth 21 and 22 measuring approximately 11 × 14 mm (Fig. 2, Fig. 3B and C)

The sensitivity tests for teeth 12, 11, 21, 22 are negative. The CBCT scan showed external resorption in tooth 21 and internal resorption in tooth 12. In tooth 21, the root canal could not be detected, indicating obliteration of the canal. Following the assessment of the radiological and clinical examinations, a treatment plan was prepared by the conservative dental specialist and oral surgeon, and the patient was informed in detail. The treatment plan included root canal treatment and root canal filling of teeth 11, 12 and 22 in the first step, followed by removal of the two cysts 48 hours later, apicoectomy of teeth 12 and 22 and filling of the cyst cavity with platelet-rich fibrin (PRF) in the second step. For the reasons detailed above, we planned the removal of tooth 21 [6].The patient did not wish to return to his previous dentist for conservative dental treatment.

## 3 Conservative dental treatment

At the first visit, after local anaesthesia (Septanest 4 %), the temporarily fixed four-unit splint was removed (Fig. 3).

**Fig. 4:**
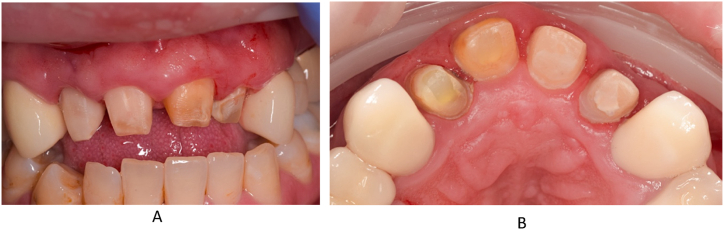
Status after the temporary bridge removal. From labial side (A) and from palatal side (B).

**Fig. 5:**
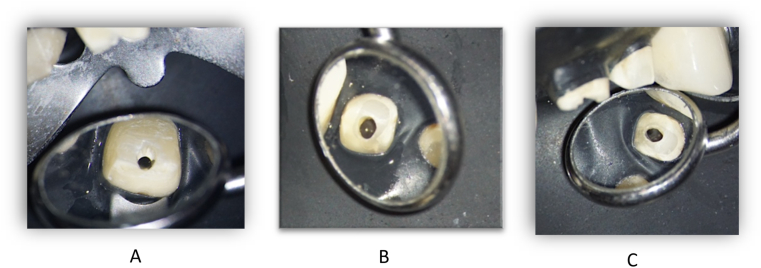
Preobturatio of the 12 (A), 11 (B) and 22 (C) teeth.

The endodontic treatment was performed under the control of an operating microscope (Labomed Prima DNT). Following the placement of the rubber dam isolation, the access cavity of tooth 11 was created and trephination was carried out. During the exploration of the root canal of the tooth, no bleeding but pulp necrosis was observed and after excision of the pulp, the canal was prepared by manual and mechanical devices. The working length was determined using an electric apex locator (Woodpecker Woodpex III). The initial file (IAF) was a manual ISO 010 (VDW C-Pilot) steel tool, the apical master file (MAF) was a 025/.08 mechanical NiTi expander (VDW Reciproc Blue R25). Fig. 4A shows the condition of the root canal of tooth 11 before preparation. The chemomechanical preparation of the root canal of tooth 11 was carried out using a crown-down technique, irrigation with sodium hypochlorite (Cerkamed Chloraxid 2 %) and physiological saline (B. Braun NaCl .9 %). The irrigation agents were activated with an ultrasonic device (Eighteeth Ultra X). The canal was dried with an Endo-Aspirator (Cerkamed) and paper points measured to working length. Following this tooth 12 was treated in the same way as described above. Due to the extensive internal resorption of the canal, the initial file was ISO 080 and the apical master file ISO 110 steel hand tool (VDW K-File). Fig. 4B shows the condition of the root canal of tooth 12 before machining. Machining was performed using a step-back technique with irrigation with 5.25 % sodium hypochlorite (Cerkamed Chloraxid 5.25 %), chlorhexidine (Cerkamed Gluco-Chex 2 %) and physiological saline. Finally, tooth 22 was treated, with the root canal treatment having been started earlier and the canal was sealed with a temporary material. After removing the temporary material from the root canal of tooth 22, the access cavity was cleaned out and the channel was made penetrable, and the working length was determined using an electric apex locator. Following excavation, large amounts of inflammatory exudate were discharged from the canal. Manual steel tools (IAF: ISO 025 C-Pilot, VDW) and mechanical expanders (MAF: 050/.05 Reciproc Blue R50, VDW) were used for the machining. Irrigation protocol was similar to the previous tooth with NaOCl 5.25 %, CHX 2 %, and physiological saline, activation with UH device 30 sec/canal. Fig. 4C shows the condition of the root canal of tooth 22 before machining. After a final canal drying, all three teeth were sealed with calcium hydroxide (Cerkamed Calcipast), the inlet cavities were temporarily sealed with a temporary filling and finally the upper four-part PMMA splint was temporarily fixed (see Fig. 5).

In a second visit, after a similar preparation as described above (local anaesthesia, removal of the temporary prosthesis, absolute isolation), the canals were rinsed according to the irrigation protocol and Ca(OH)_2_ was replaced. There was still some exudate from tooth 22 through the orifice, although less than the first time.

**Fig. 6:**
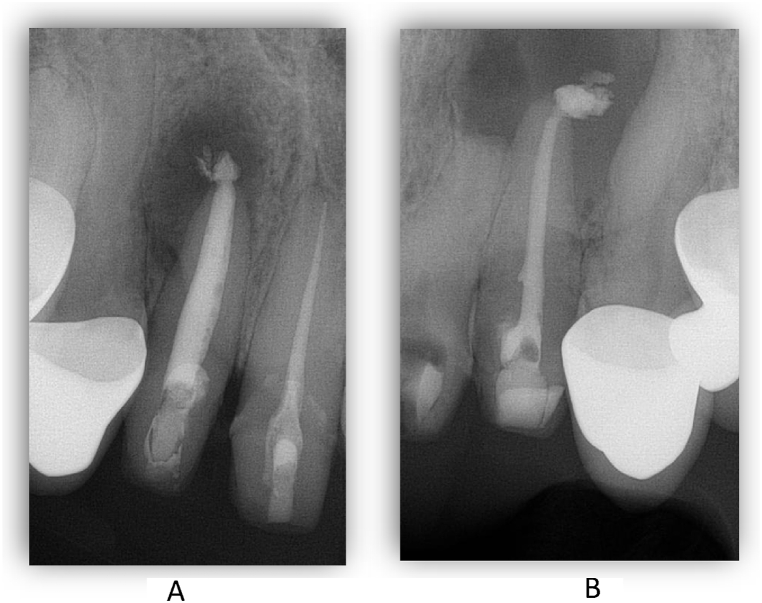
Periapical X-rays after the rootcanal treatments. 12 and 11 teeth (A) and the 22 tooth (B).

Completing the endodontic treatment was planned for the third visit. After preparation, final irrigation of the root canals was performed using 2 % (tooth 11) or 5.25 % (teeth 12, 22) sodium hypochlorite, 17 % EDTA (Cerkamed Endo Solution), 2 % chlorhexidine, physiological saline at 5 ml per canal and ultrasonic stimulation as described above. Lateral condensation was used as root canal filling technique for tooth 12 and a modified single-cone (isometric gutta-percha) technique was used for teeth 11 and 22. For all three teeth, an injectable tricalcium silicate-based bioceramic sealer (FKG TotalFill BC Sealer) was used. After the root canal fillings were made, the access cavities were closed with glass ionomer cement (GC Fuji IX) and a postoperative control radiographs were taken (Fig. 6).

## 4 Oral surgical treatment

**Fig. 7:**
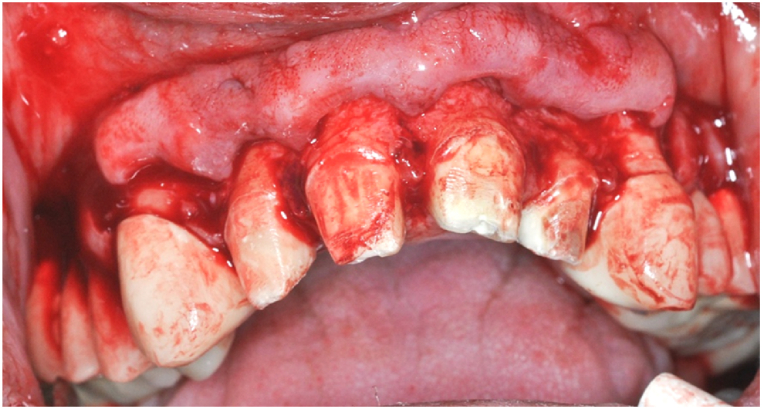
Wassmund flap preparation.

**Fig. 8:**
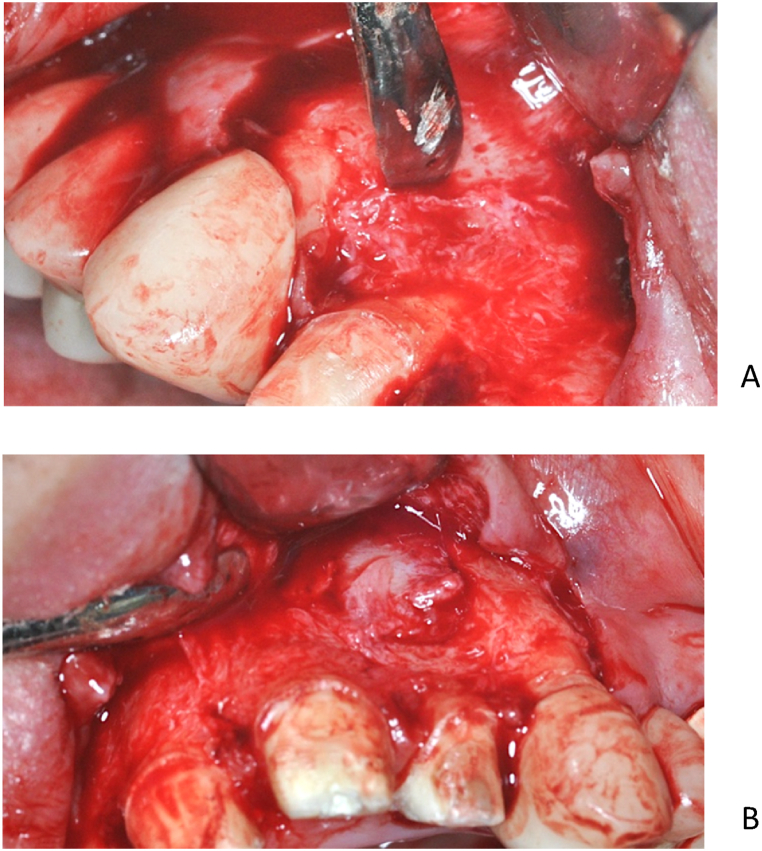
The spontanous fenestration of the labial cortical bone on the right (A) and left (B) side.

Oral surgery was performed 48 hours after the root canal fillings were made. After local anaesthesia (Ultracain DS Forte 4 %), a Wassmund's mucoperiosteal flap was formed. The verical incisions were made at teeth 13 and 23 (Fig. 7) Following flap preparation, fenestrations were seen in the labial cortical bone of both cysts (Fig. 8A and B).

**Fig. 9:**
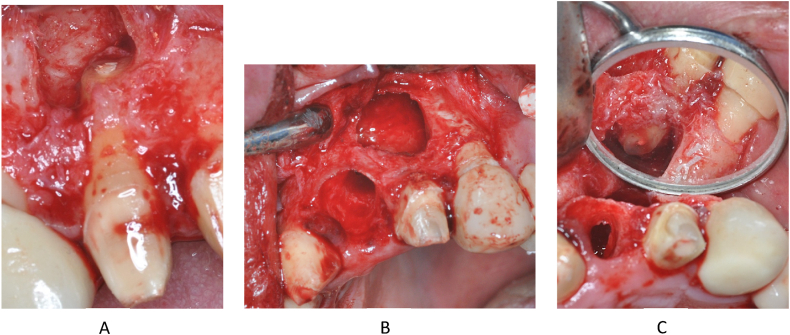
The apicoectomy of the 12 tooth (A). Status after the 21 tooth extraction and cystectomy ont he left side (B). The apicoectomy of the 22 tooth (C).

On the right side, starting from the fenestration, the labial cortical window was enlarged with a surgical spherical bur. The cystic epithelium was then removed and the apex of the root of tooth 12 was amputated (Fig. 9A). After removal of the cyst, no permeability to the palate or nasal cavity was observed, the cyst cavity was bordered by a bony wall on all surfaces. In the situation of the left cyst because of the large bone perforation, it was not necessary to significantly increase the bone window on the labial cortical. After dissection and removal of the cystic epithelium, tooth 21 (Fig. 9B) was removed as planned and on tooth 22 apicoectomy was carried out. (Fig. 9C).

**Fig. 10:**
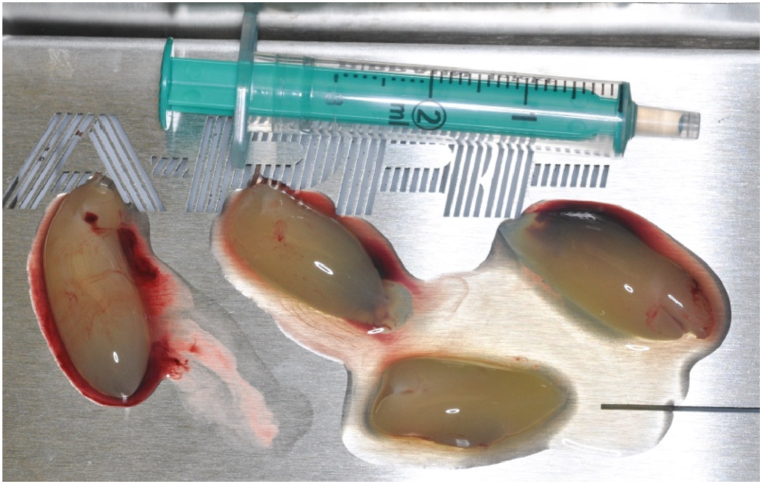
Making of the PRF clots.

**Fig. 11:**
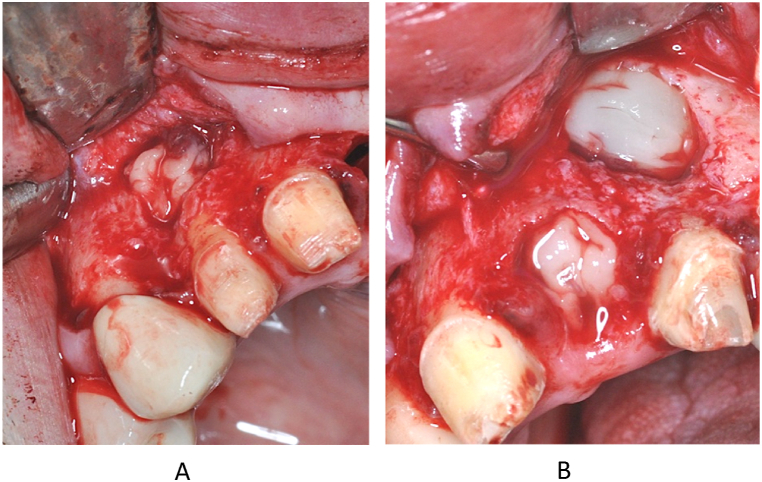
PRF clots placed into the bone cavity on the right (A) and left (B) side.

On inspection of the cystic cavity towards the base of the nasal cavity and towards the palate, no bony wall was detected, but no mucosal perforation was found. Considering the newly prepared lege artis bioceramic orthograde root canal fillings in the resected teeth, the preparation of retrograde root fillings was not necessary. After smoothing out the sharp edges of the bone windows, the cyst cavity was rinsed with physiological saline. In the next step, intravenous blood was collected from the patient in tubes without anticoagulant (A-PRF + tube) and immediately placed in the centrifuge (1300 rpm, 8 min) according to the Choukroun protocol [7] The appropriate fraction was then separated and PRF clots and membranes were generated (Fig. 10). The resulting PRF clots were used to fill the cyst cavities and the alveoli after extractions, and the area was then covered with mebranes (Fig. 11).

**Fig. 12:**
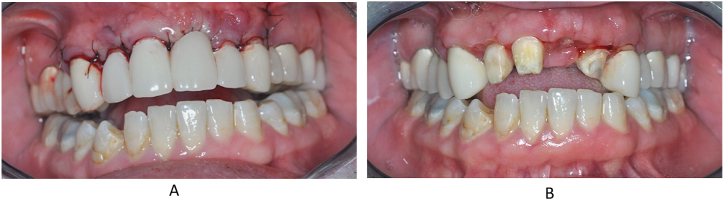
The sutured wounds and the replaced temporary bridge after surgery procedure (A) and the status on the first week control (B).

**Fig. 13:**
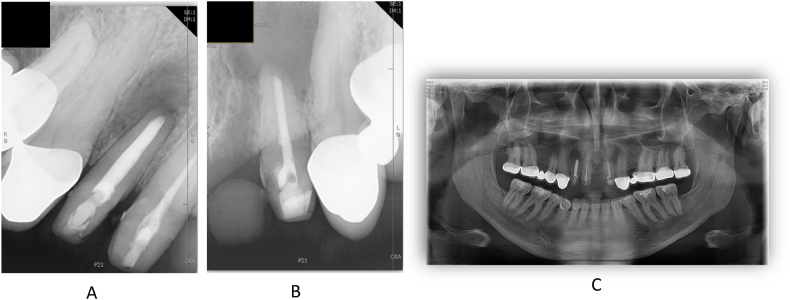
Postoperativ periapical x-ray about the 12 tooth (A), about the 22 tooth (B) and the orthopantomogram (C).

Subsequently, the wound was closed with Supramid 5/0 thread per primary simple knot and vertical mat stitches according to the incision lines and adapted at the extraction alveolus of tooth 21. The patient's temporary restoration was cemented back immediately after the surgery procedure. (Fig. 12A). The sutures were removed 1 week after the operation. (Fig. 12B). Periapical control radiographs were taken on the day of surgery (Fig. 13).

**Fig. 14:**
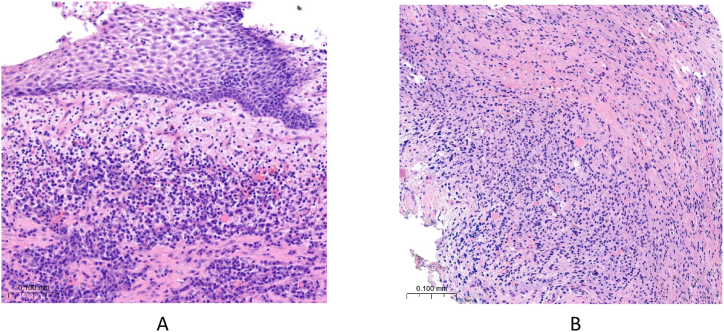
Histological section of the sample taken from the right and left cyst with HE staining at 20x magnification.

After surgery, clindamycin 300 mg 4x1 and diclofenac 50 mg 3x1 therapy was started. Suture removal was performed after 1 week: the postoperative period was uneventful, wound healing was physiologically adequate. The cysts were sent for histological examination, which showed a radicular cyst on both sides. Histological sections of the right cyst in Fig. 14A and the left cyst in Fig. 14B are shown at 20x magnification with hematoxylin-eosin (HE) staining.

**Fig. 15:**
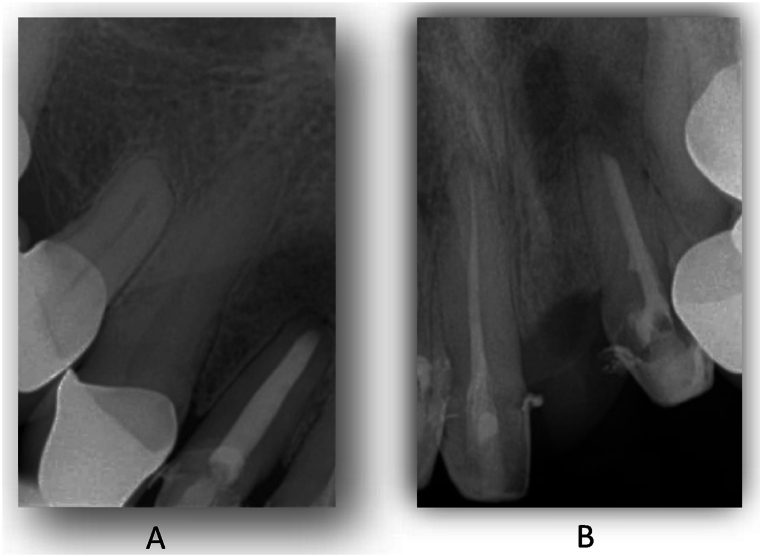
Periapical X-ray about the 12 tooth (A) and about the 11 and 22 teeth (B) after 3 mounth of the surgical procedure.

**Fig. 16:**
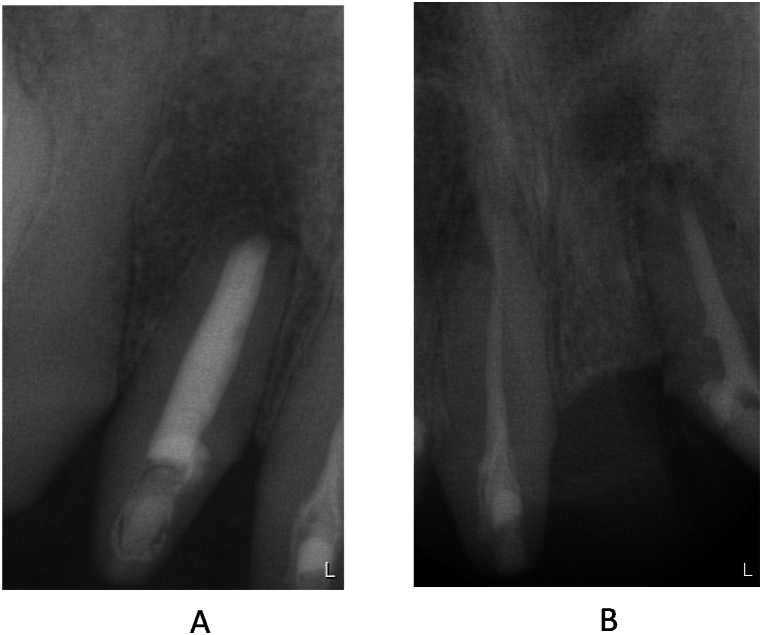
Periapical X-ray about the 12 tooth (A) and about the 11 and 22 teeth (B) after 31 month of the surgical procedure.

**Fig. 17:**
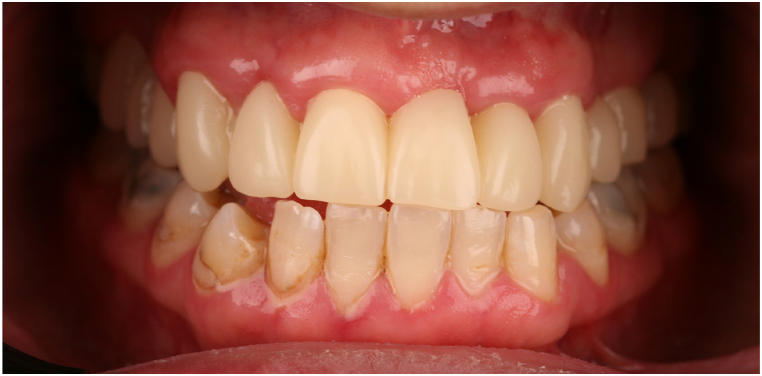
The status after the prosthetic rehabilitation.

After the operation and the suture removal, the patient unexpectedly moved abroad, so we were unable to follow up. However, the patient visited a dentist at his new place of residence and sent us the periapical X-rays he had taken. The radiographs were taken 4 months after surgery and clearly show that the large cysts had almost completely regenerated (Fig. 15). In the autumn of 2023, the patient came back to us to continue his dental rehabilitation. The radiographs taken demonstrate that after 31 months, the healed condition is stable and the periapical region of the affected areas is intact (Fig. 16). The patient's final prosthetical rehabilitation on the upper jaw was carried out with a zirconium bridge (Fig. 17).

## 5 Discussion

In our case, both the conservative dental treatments and the oral surgical procedures were performed using the latest materials and technologies currently available. The irrigation protocol described in the endodontic treatment is based on literature recommendations [8,9]. The dose concentration of NaOCl, the combination of irrigants and the intracanal medication used were based on the clinical diagnosis of the tooth [[10], [11], [12]]. The choice of sealer material for obturation was based on several aspects of the literature published in recent years. TotalFill BC Sealer is made up of 35–45 % zirconium oxide, 20–35 % tricalcium silicate, 7–15 % dicalcium silicate and 1–4% calcium hydroxide. The antimicrobial properties of the so-called bioceramic sealer group, as well as its apical sealing, have been shown to be superior to gold-standard epoxy resin (AH Plus, Dentsply) or eugenol-based (Endomethasone N, Septodont) root canal fillers in both non-surgical and surgical treatments [13,14]. The benefits of the sealer properties (hydrophilicity, bond expansion) have been clinically proven to be excellent for root sealing techniques combined with isometric gutta-percha [15]. Its production by nanoparticle technology, in addition to chemomechanical machining, provides a complete hermetic seal throughout the root canal system [16].

The use of various blood products and bone substitutes in the modern treatment of jawbone cysts is now well documented [[17], [18], [19], [20], [21]]. In addition to platelet-rich plasma (PRF) and platelet-rich growth factor (PRGF), platelet-rich fibrin (PRF) is now the most widely used blood products [22,23]. The method for the production of PRF was developed by Choukroun [24] in 2001. This material is a second generation platelet concentrate, which is used to obtain a fibrin membrane rich in platelets, leukocytes and growth factors, free of any additives and therefore fully autologous. PRF forms a strong, natural fibrin matrix with a complex structure and mechanical properties unmatched by other platelet concentrates. Leukocytes and growth factors (Platelet-derived Growth Factor - PDGF, Transforming Growth Factor - TGF-a, Transforming Growth Factor - TGF-b, Insulin-like Growth Factor - IGF-I) embedded and concentrated in fibrin are released gradually, prolonging the effect over time. It has been proven to promote the proliferation of osteoblast, fibroblast, pulp and periodontal ligament cells, while suppressing the migration of epithelial cells, thus promoting tissue regeneration [7,17,23,25,26]. When larger cysts are removed in toto, secondary infection and various wound healing disorders are common due to coagulum retraction. The alternative is to have a cystostomy and obturator made and then removed in a second operation. However, this means more surgery, prolonged recovery time, more treatment sessions and a great deal of discomfort for patients. The time needed for full recovery can be delayed by years. The use of PRF in this field gives us the opportunity to remove larger cysts in toto, as the inserted fibrin clot forms a stable matrix, thus eliminating complications from retraction, and the growth factors released accelerate bone regeneration and promote healing, as well as reduce postoperative complaints [7,19,20,27,28]. There is no consensus in the literature on the use of bone graft substitutes in the treatment of jaw bone cysts. Some recommend the combined use of blood products and bone substitutes, while others prefer the use of blood products alone. In the case of autologous bone substitutes, the morbidity of the donor site, more postoperative pain and higher resorption rates are disadvantages, while in the case of xenogeneic materials, the significant cost, foreign body reactions and potential infections may be a disadvantage. We have chosen to use PRF alone for reasons of minimal invasiveness, cost-effectiveness and successful application as widely described in the literature [21,22,26,29]. In our case, due to careful treatment, the cysts were almost completely healed in 4 months and the successful result has shown a stable condition for 2.5 years. The prosthetic rehabilitation could have started a few months after the surgery, so we could have provided a short treatment period. However, for the reasons mentioned above, this has not happened until now.

## 6 Conslusion

Based on this case, it can be said that interdisciplinary cooperation and knowledge of rapidly evolving dental materials and devices are essential for professionals in the dental care of patients today. In this way, we can ensure that teeth previously planned for removal remain functional, or that previously time-consuming treatments are significantly reduced. In our case, we demonstrated this through the treatment of odontogenic cysts, but the statement can also be true for many other dental conditions. The rapid development of dentistry and dental materials makes it essential for doctors in each speciality to continue their training at the same pace in order to provide state-of-the-art patient care.

## Data availability statement

No data was used for the research described in the article.

## CRediT authorship contribution statement

**Kinga Bérczy:** Writing – review & editing, Writing – original draft. **Csilla Erdei:** Formal analysis. **Hajnalka Rajnai:** Investigation. **Gergely Hriczó-Koperdák:** Investigation. **Árpád Fancsaly-Joób:** Conceptualization. **Noémi Kovács:** Investigation, Supervision.

## Declaration of competing interest

The authors declare that they have no known competing financial interests or personal relationships that could have appeared to influence the work reported in this paper.
